# Spatial patterns of the frog *Oophaga pumilio* in a plantation system are consistent with conspecific attraction

**DOI:** 10.1002/ece3.3748

**Published:** 2018-02-14

**Authors:** Brian Folt, Maureen A. Donnelly, Craig Guyer

**Affiliations:** ^1^ Department of Biological Sciences and Auburn University Museum of Natural History Auburn University Auburn AL USA; ^2^ Department of Biological Sciences Florida International University Miami FL USA

**Keywords:** colonization, habitat selection, nearest‐neighbor analysis, neotropics, spatial ecology

## Abstract

The conspecific attraction hypothesis predicts that individuals are attracted to conspecifics because conspecifics may be cues to quality habitat and/or colonists may benefit from living in aggregations. Poison frogs (Dendrobatidae) are aposematic, territorial, and visually oriented—three characteristics which make dendrobatids an appropriate model to test for conspecific attraction. In this study, we tested this hypothesis using an extensive mark‐recapture dataset of the strawberry poison frog (*Oophaga pumilio*) from La Selva Biological Station, Costa Rica. Data were collected from replicate populations in a relatively homogenous *Theobroma cacao* plantation, which provided a unique opportunity to test how conspecifics influence the spatial ecology of migrants in a controlled habitat with homogenous structure. We predicted that (1) individuals entering a population would aggregate with resident adults, (2) migrants would share sites with residents at a greater frequency than expected by chance, and (3) migrant home ranges would have shorter nearest‐neighbor distances (NND) to residents than expected by chance. The results were consistent with these three predictions: Relative to random simulations, we observed significant aggregation, home‐range overlap, and NND distribution functions in four, five, and six, respectively, of the six migrant–resident groups analyzed. Conspecific attraction may benefit migrant *O. pumilio* by providing cues to suitable home sites and/or increasing the potential for social interactions with conspecifics; if true, these benefits should outweigh the negative effects of other factors associated with aggregation. The observed aggregation between migrant and resident *O. pumilio* is consistent with conspecific attraction in dendrobatid frogs, and our study provides rare support from a field setting that conspecific attraction may be a relevant mechanism for models of anuran spatial ecology.

## 1 INTRODUCTION

A controversial factor influencing habitat selection is the role that individuals play in shaping the distribution of conspecifics. A large body of literature posits that, because conspecifics are competitors, individual fitness should decline with increasing conspecific density (Brown, 1969; Muller, Stamps, Krishnan, & Willits, 1997; Rosenzweig, 1985, 1991). This theory predicts that, to minimize intraspecific competition, individuals seeking habitat should avoid areas with established conspecifics. However, a contrary line of evidence suggests that individuals can be attracted to conspecifics (conspecific attraction; Stamps, 1988), because (1) conspecifics serve as cues of habitat quality (conspecific cueing; Stamps, 1987), (2) colonists may benefit from living in aggregations after territories are established because individuals in aggregations may better protect territories, reduce predation, and promote social interaction or attract mates (Boulinier & Danchin, 1997; Muller et al., 1997; Stamps, 1988, 1994), and/or (3) individuals may reduce costs associated with prospection (Reed, Boulinier, Danchin, & Oring, 1999). Conspecific attraction is particularly applicable to: (1) territorial species, because presence of territorial residents may indicate that a habitat is of sufficient quality to justify occupancy and defense (Stamps, 1987) and (2) aposematic species, because individuals living in close proximity increase the strength of aposematic signals (Sillén‐Tullberg & Leimar, 1988). In the conspecific attraction model, the probability of settlement is increased in the presence of conspecifics (Donahue, 2006). For conspecific attraction to be an adaptive strategy, fitness increases accrued from a settlement with conspecifics must outweigh the energetic costs associated with higher densities and increased intraspecific competition. Thus, conspecific attraction predicts that individuals with little or no experience should be more attracted to habitat with a higher density of conspecifics than other individuals already experienced with the habitat (Donahue, 2006; Stamps, 1988).

A recent review of reptile social behavior emphasizes the importance of and need for more studies of conspecific attraction (Doody, Burghardt, & Dinets, 2013). Despite much interest in conspecific attraction by population and conservation biologists (e.g., Campomizzi et al., 2008; Fletcher, 2006; Lima & Zollner, 1996; Ward & Schlossberg, 2004), empirical studies of whether conspecific attraction influences selection are logistically challenging, because it can be difficult to control for habitat quality in natural heterogeneous landscapes (Stamps, 1988). However, support for conspecific attraction has been found for invertebrates (Crisp, 1976; Donahue, 2006; Meadows & Campbell, 1972; Muller, 1998), fish (Sweatman, 1985, 1988), amphibians (Gautier, Olgun, Uzum, & Miaud, 2006; Pizzatto et al., 2015), reptiles (Clark, 2007; Stamps, 1987, 1988), and birds (Muller et al., 1997; Danchin, Boulinier, & Massot, 1998; Etterson 2003; Ward & Schlossberg, 2004; Austin, Neil, & Warren, 2017). Together, these studies suggest conspecific attraction may be a pervasive mechanism among diverse animal groups.

Within amphibians, few studies have tested for evidence of conspecific attraction (Gautier et al., 2006; Gonzalo, Cabido, Galán, López, & Martín, 2006; Pizzatto et al., 2015), but there is good reason to suspect that conspecific attraction might be an important mechanism influencing the spatial ecology of frogs, particularly for poison frogs in the family Dendrobatidae. The single study supporting conspecific attraction in anurans suggested it may be mediated by visual cues (Pizzatto et al., 2015). Dendrobatids are diurnal frogs that both use visual and acoustic cues during mate choice (Narins, Hödl, & Grabul, 2003; Reynolds & Fitzpatrick, 2007; Summers, Symula, Clough, & Cronin, 1999) and parental care (Stynoski & Noble, 2012). Further, many dendrobatids are highly territorial: Males vigorously defend areas with suitable sites for advertisement, courtship, and oviposition (Donnelly, 1989a; Pröhl & Hödl, 1999; Roithmair, 1992, 1994). Last, many poison frog species are aposematic (e.g., Saporito, Zuercher, Roberts, Gerow, & Donnelly, 2007; Saporito, Donnelly, et al., 2007), an evolutionary feature that benefits from clustering among individuals (Sillén‐Tullberg & Leimar, 1988). Thus, the high visual orientation, territoriality, and aposematism make dendrobatid frogs an ideal candidate group to test whether conspecific attraction influences habitat selection of frogs in diverse lowland Neotropical forests.

The conspecific attraction hypothesis predicts that, across habitats of equivalent quality, naïve juveniles and migrating adults will preferentially colonize and associate in space with preestablished adults more frequently than expected by chance. Here, we tested this prediction using a large mark‐recapture dataset of a territorial and aposematic dendrobatid frog, *Oophaga pumilio,* at La Selva Biological Station, Costa Rica. We sampled *O. pumilio* populations in replicate plots within an abandoned *Theobroma cacao* plantation. We used the cacao plantation system because leaf litter and trees were uniformly distributed throughout, which allowed us to establish replicate plots of relatively homogenous habitat quality (leaf litter, bromeliads) for this and other studies (Donnelly, 1989a,b; Guyer, 1988a,b). We made three predictions about the spatial distribution of *O. pumilio* to test the conspecific attraction hypothesis: (1) individuals entering a population (i.e., demographic recruitment from births [juveniles] or migrating adults, hereafter, collectively “migrants”) would aggregate around resident adults rather than distributing themselves in a random or uniform distribution relative to residents, (2) migrants would share sites with residents at a higher frequency than expected by chance, and (3) home‐range locations of migrants and residents would have nearest‐neighbor distances consistent with aggregation.

## 2 MATERIALS AND METHODS

### 2.1 Study site and species

La Selva Biological Station (hereafter, La Selva) is a private reserve owned by the Organization for Tropical Studies (OTS) in the Caribbean lowlands of northeastern Costa Rica, ca. 3 km south of Puerto Viejo de Sarapiquí, Heredia Province (10.42°N, 84.02°W). Elevation at La Selva ranges from 30 to 130 m asl. The site is characterized by an average temperature of 25.8°C, receives ca. 4 m of precipitation per year (Sanford, Paaby, Luvall, & Phillips, 1994), and is classified within Holdridge's Tropical Wet Forest life zone (McDade & Hartshorn, 1994). Rainfall is seasonal with the most rain occurring during the wet season (May–December), relative to the dry season (January–April).

**Figure 1:**
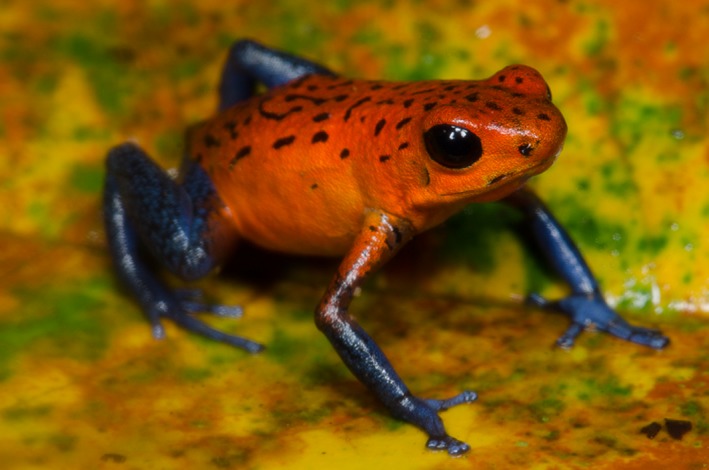
The strawberry poison frog (*Oophaga pumilio*) from La Selva Biological Station, Costa Rica. Photograph by J. Folt

*Oophaga pumilio* (strawberry poison frog; Figure 1) is an abundant species of poison frog (family Dendrobatidae) occupying terrestrial habitats in lowland Caribbean forests from Nicaragua to Panama. The species is a dietary specialist consuming ants and mites (Donnelly, 1991); these taxa, which compose >80% of its diet, are sources of alkaloid compounds that are sequestered into poison glands in the frog's skin (Saporito et al., 2004; Saporito, Zuercher, et al., 2007), providing a chemical defense from predators (e.g., Stynoski, Torres‐Mendoza, Sasa‐Marin, & Saporito, 2014). Across its geographic distribution, the species is brightly colored, which is an aposematic signal to predators (Saporito, Donnelly, et al., 2007). Both sexes provide parental care. Fathers guard and hydrate fertilized eggs in leaf litter, and mothers transport hatched tadpoles to rearing sites in bromeliads (Weygoldt, 1980). Tadpole‐rearing sites are repeatedly revisited by mothers to provision tadpoles with unfertilized eggs (Brust, 1993), which provide nutrition for growth and alkaloids for chemical defense (Stynoski et al., 2014). Both sexes can be territorial: Whereas females have been found to defend foraging areas (Meuche, Linsenmair, & Pröhl, 2011), males are more strongly territorial, defending areas with suitable sites for advertisement, courtship, and oviposition (Pröhl & Hödl, 1999). Bromeliad availability has also been experimentally demonstrated as a limiting resource that regulates the abundance of males, which may actively defend those resources (Donnelly, 1989a). Territorial males attempt to attract females by perching in elevated sites and advertising with vocalizations; these individuals usually are large and can produce calls with low dominant frequencies to deter rivals (Meuche, Linsenmair, & Pröhl, 2012). However, some males use alternative, noncalling mating tactics to parasitize advertising territorial males (i.e., satellite males; Meuche & Pröhl, 2011). Home‐range size of females is larger than that of males (Donnelly, 1989b; Pröhl & Hödl, 1999; Savage, 2002), is independent of density (Donnelly, 1989b), and may, in part, provide females access to bromeliads and mates (Murasaki, 2010). The species’ mating system has been described as sequential polygamy comprising sequential and simultaneous polygyny and sequential polyandry (Pröhl & Hödl, 1999).

### 2.2 Data collection

The study site was an abandoned *Theobroma cacao* (hereafter, cacao) plantation system at La Selva. The plantation was dominated by cacao, but also contained *Bactris gasipaes* (pejibaye; peach palm) and *Cordia alliodora* (laurel; a hardwood) interspersed at regular intervals. The cacao trees were spaced at regular, 3‐m intervals at planting, and created a simple and regular environment, even with intermittent taller laurel and pejibaye penetrating the cacao canopy. The plantation activities ended in 1963 when the Organization for Tropical Studies (OTS) acquired the property. We used the cacao system here because plantations generate homogenous terrestrial habitats, which provide environmental controls that cannot be found in natural forests (Boucher, Hansen, Risch, & Vandemeer, 1983). Thus, our rationale for using this site was similar to that of other projects, which sought to take advantage of the relatively simple understory and homogeneity of the cacao‐dominated environment to control for habitat variability while examining how other features influence population ecology of terrestrial vertebrates (e.g., Donnelly, 1989a,b; Guyer, 1988a,b).

To this end, we established four gridded plots (12 m × 9 m) within the cacao system, with individual cacao trees providing a symmetrical grid system of forty‐eight individually identifiable 1.5 m × 1.5 m cells within each plot. Plots were separated by 25 m. From February 1982 to August 1983, *O. pumilio* were surveyed diurnally in each plot 2–10 times per month (mean = 3.5) using capture‐mark‐recapture techniques (Donnelly & Guyer, 1994). Plots were surveyed by walking in a zig‐zag pattern through tree rows, searching for individuals active on the surface of leaf litter or understory vegetation up to 2 m above the ground. Captured individuals were measured for snout‐vent length (SVL; mm) and mass (g) and were classified into two age‐class groups: juveniles (<19 mm snout‐vent length [SVL]) and adults (≥19 mm SVL). Adults were further identified as male (presence of a darkly pigmented gular sac) or female (possessing red throat coloration; Bunnell, 1973; Donnelly, 1989c), and capture location was recorded as within a particular grid cell. Each individual was assigned a unique combination of toe clips and marked accordingly to facilitate individual identification during recaptures. When juveniles were recaptured and measured to a size of ≥19 mm SVL, individuals were considered to have matured and were categorized as male or female. Each plot was surveyed ten times in April 1982 to obtain a relatively accurate estimate of the number of individuals present in plots; in most other months, plots were surveyed 3–4 times/month. The study period encompassed seasonal replication of dry and wet seasons (*N* = 2, respectively); however, weather was characterized by an El Niño event such that weather conditions were more strongly seasonal than usual for La Selva (e.g., see Guyer, 1988a).

### 2.3 Statistical analysis

Capture histories were combined into three–four‐month intervals throughout the sampling period. Months were pooled to capture seasonal variation in climate and rainfall: the dry season in 1982 (February–May), the first and second half of the wet season spanning 1982 into 1983 (June–September; October–January), the dry season in 1983 (February–May), and the start of the wet season in 1983 (June–August). Individuals were classified as migrants or residents within each plot on the basis of size (an estimate of age) and apparent duration of their presence in a given plot. For adults, we classified individuals as residents if they had been observed in a given plot during the previous season. We considered all juveniles as migrants because they recently entered into the population and had relatively little experience in the habitat they occupied, features similar to individuals migrating to novel habitat. Upon reaching maturity and transitioning to the adult stage class, individuals were classified as residents if they remained within the same plot. This classification system resulted in individuals being labeled as resident adults (resident females [RF], resident males [RM]) and recently migrated adults (migrant females [MF], migrant males [MM]), and juveniles (J). As our criteria for classifying migrants and residents were unable to determine the status of adults in the first season, all adults were classified as residents.

We used two versions of the dataset in the analyses: (1) a dataset including all observations of every individual recorded in the four plots (hereafter, full dataset) and (2) a dataset restricted to include individuals captured ≥ three times (hereafter, subsetted dataset). The subsetted dataset was used to remove individuals whose tenure in plots was brief (i.e., temporary, nonresident visitors) and was used to elucidate spatial distribution among migrating individuals and residents.

We described seasonal variation in abundance by measuring population structure of *O. pumilio*, and we used the subsetted dataset to calculate the mean number of observed juveniles, females, and males across each plot. We developed five a priori candidate models to explain variation in abundance: (1) null model, (2) seasonal variation, (3) variation by age‐sex classes (juvenile, female, male groups), (4) group and seasonal variation, and (5) a saturated (full) model with variation by group, season, and a group–season interaction. We used the candidate models to guide construction of linear mixed‐effects models explaining variation in observed abundance, with plot assigned as a random effect. We ranked models using Akaike's information criterion adjusted for small sample sizes (AIC_c_; Hurvich & Tsai, 1989), and we used model weight statistic to measure the probability a given model represented the true best model among all candidates (Burnham & Anderson, 2002).

To test predictions of the conspecific attraction hypothesis, we analyzed the spatial arrangement of individuals within populations (i.e., second‐order habitat selection; Johnson, 1980). First, we used the subsetted dataset and averaged the two‐dimensional capture coordinates for each individual to estimate a mean centroid of space use during each season. We then calculated Clark and Evans (1954) *R*‐value, an index of spatial dispersion that measures the degree to which individuals exhibit a clumped (*R *<* *1.00), random (*R* ~ 1.00), or uniform (*R *>* *1.00) distribution (Clark & Evans, 1954; Krebs, 1999) at the seasonal scale. We measured the *R*‐value with border correction (Donnelly, 1978) for juveniles, migrating females, and migrating males relative to resident females and resident males during each season in each plot.

Next, we sought to analyze space use and co‐occurrence patterns at shorter intervals by measuring shared sites between age and sex groups. We estimated the observed proportion of shared sites (1.5 m × 1.5 m grid cells) by dividing the number of sites occupied by ≥ two individuals of different migrant–resident classes during each survey by the total number of sites occupied. The proportion of shared sites was measured for the same groups as in the *R*‐value analysis using both the full and subsetted datasets.

**Figure 2:**
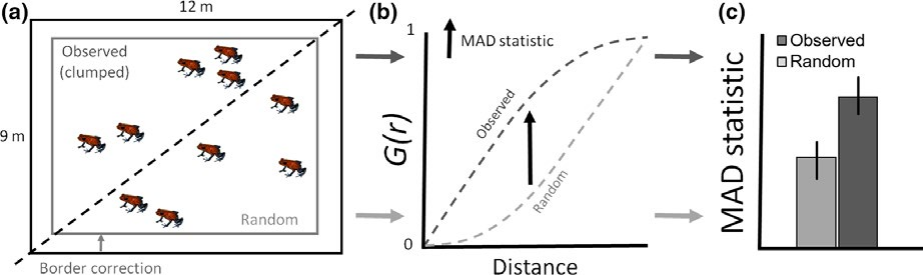
A conceptual model describing the analytical framework used to evaluate nearest‐neighbor distances of *Oophaga pumilio* in cacao plantation at La Selva, Costa Rica. (a) A plot in our study system illustrating two hypothetical spatial distributions of individuals (separated by black hash): Five individuals that are observed to be clustered (top‐left), and five individuals that are randomly distributed (bottom‐right). The outer black box indicates the study area of plots (12 m × 9 m); the inner,gray box indicates a border correction used by our analyses (not to scale). (b) A hypothetical graph describing nearest‐neighbor distance distribution functions *G*(*r*) for clustered nearest neighbors (dark gray hash) and randomly distributed neighbors (light gray hash). The black arrow indicates a deviation between clustered and random neighbors, as measured by the maximum absolute deviation test statistic (MAD statistic). (c) Bar plots describing mean MAD test statistics (with confidence intervals) between observed distances of clustered individuals (dark gray) and those from randomly distributed data (light gray)

Last, we sought to examine nearest‐neighbor distances (NND; Clark & Evans, 1954) among individuals of the migrant–resident groups. Because classic NND can be confounded when true nearest neighbors do not occur within the study area and/or when the study area is irregular in geometry (Cressie, 1991), we used an analysis which accounts for edge effects and irregular geometry, the nearest‐neighbor distance distribution function *G*(*r*) (Cressie, 1991). *G*(*r*) estimates how the density of a static point process increases with distance from a focal point, given complete spatial randomness (CSR), and then compares the random pattern to that of an observed NND distribution function, *Ĝ*(*r*). Analyses where *Ĝ*(*r*) > *G*(*r*) indicate that nearest‐neighbor distances in the observed pattern are shorter than predicted by a random process and suggest clustering. Conversely, *Ĝ*(*r*) < *G*(*r*) indicates greater distances among points than expected by random, a uniform pattern. We derived a mean centroid of space used for all individuals in each pooled sample of months using the subsetted dataset and estimated *G*(*r*) and *Ĝ*(*r*) for the same migrant–resident classes as in the *R*‐value analysis. Border correction was implemented using the spatial Kaplan–Meier estimator (Baddeley & Gill, 1997). To analyze whether individuals exhibited aggregation in all seasons and plots, we performed the maximum absolute deviation test (MAD test; Diggle, 1986; Cressie, 1991; Loosmore & Ford, 2006) with one tail and used the MAD test statistic as a proxy for aggregation among individuals. We describe our conceptual framework for the nearest‐neighbor distance analyses in Figure 2.

To determine if observed spatial distribution patterns deviated from those expected by CSR, we estimated *R*‐values, proportion of shared sites, and *G*(*r*) for randomly generated distributions of individuals. Simulated distributions were generated using the same density of migrant–resident classes observed in plots during each sampling unit (season for *R‐*values and *G*(*r*); individual surveys for proportion of shared sites). Because mean random values varied among simulations, we performed replicate simulations (*N* = 10) to better approximate true random means. We tested if observed patterns differed from those expected by chance using a paired linear mixed‐effect models; to account for some seasonal differences in abundance, season was nested within plot as a random effect in all mixed‐effect models. We used the statistical program R (Program R; R Core Team 2015) for analyses, using functions in the packages *spatstat* (Baddeley, Rubak, & Turner, 2015) and *nlme* (Pinheiro, Bates, DebRoy, & Sakar, 2016) and with α = 0.05.

Our data were collected using a mark‐recapture (MR) framework, and our analysis made use of those MR data by inferring the tenure of each individuals’ residency in plots. While this may seem like a limited use relative to more complicated MR analyses which account for imperfect detection to estimate abundance, our primary objectives were not directly focused on estimating abundance, but rather involved modeling spatial distributions. While recent analytical advances have developed spatially explicit MR analyses (e.g., Efford & Fewster, 2013) to estimate density, these models cannot use detection probability to infer individual location and thus do not provide added benefit to the current project. Therefore, we analyzed observed spatial distributions, but we acknowledge that the results may be biased toward describing spatial patterns among individuals or groups within the population characterized by greater detection probability.

Our data and R script (Folt et al.^2018^) are available from the Dryad Digital Repository: https://doi.org/10.5061/dryad.42kp3.

## 3 RESULTS

**Figure 3:**
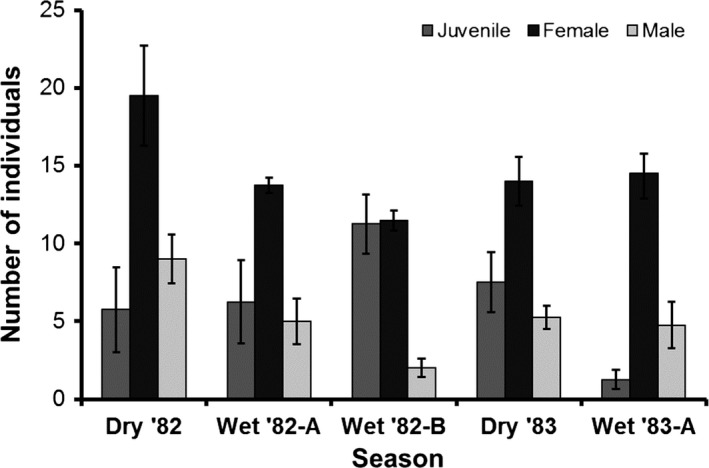
Seasonal variation in the population structure of *Oophaga pumilio* in replicate plots (*N* = 4) of *Theobroma cacao* (cacao) plantations at La Selva Biological Station, Costa Rica. Histograms represent mean (± 1 *SE*) abundances of individuals observed ≥3 times. Apparent population structure was consistent across plots, with female‐dominated adult sex ratios and comparable abundance of juveniles and adult males

The full dataset included 1661 observations of 463 individuals made during the study. Of this total, 189 individuals were captured ≥3 times (total capture = 1297); these individuals composed the subsetted dataset. Population structure was generally consistent across seasons and was characterized by strongly female‐biased sex ratios (Figure 3). The most well‐supported model identified by the model‐ranking procedure (ΔAIC_c_ = 0.00; model weight = 1.00) described abundance as a function of age‐sex groups, season, and an interaction age‐sex group and season (saturated model; Appendix S1). The model described two significant patterns: (1) juveniles and males did not differ in abundance (*p* = .18), whereas females were more abundant than both juveniles and males (*p* < .001), and (2) female and male abundance decreased in the second half of the 1982 wet season (*p* < .001, *p* < .001, respectively; Figure 3), whereas juvenile abundance increased in that season (*p* = .027; Figure 3).

**Figure 4:**
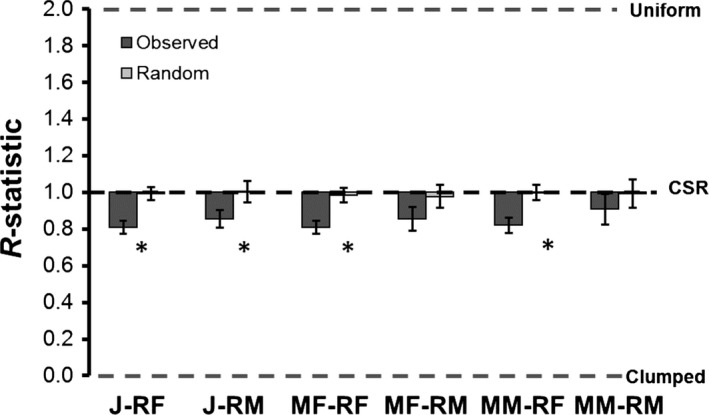
Spatial arrangement of migrant and resident *Oophaga pumilio*, as described by Clark and Evans (1954) *R*‐value (mean ± *SE*) with border correction, in a cacao plantation environment at La Selva, Costa Rica. Values <1.00 indicate a clumped distribution pattern (bottom gray hash), values ~1.00 indicate a random distribution (complete spatial randomness [CSR]—black hash), and values >1.00 indicate a uniform distribution pattern (top gray hash). Groups are juveniles with resident females (J‐RF) and resident males (J‐RM), migrant females with resident females (MF‐RF) and resident males (MF‐RM), and migrant males with resident females (MM‐RF) and resident males (MM‐RM). Asterisks (*) indicate groups that deviated significantly from complete spatial randomness (CSR; black hashed line)

We observed a significantly clumped spatial distribution in four of the six migrant–resident groups examined (Figure 4). Observed *R*‐values for juveniles with resident females (0.81 ± 0.04 *SE*) and resident males (0.86 ± 0.05 *SE*) were both more clumped than that expected by chance (*p* < .001, *p* = .032, respectively). Migrant females and migrant males were clumped with resident females (0.81 ± 0.04, *p* = .006; 0.82 ± 0.04, *p* < .001; respectively), whereas both migrant females and migrant males did not clump with resident males (0.87 ± 0.07, *p* = .12; 0.93 ± 0.08, *p* = .55, respectively, Figure 4). An overall test for differences in *R*‐values among the six groups was not significant (*F*_5,107_ = 0.71, *p* = .62).

**Figure 5:**
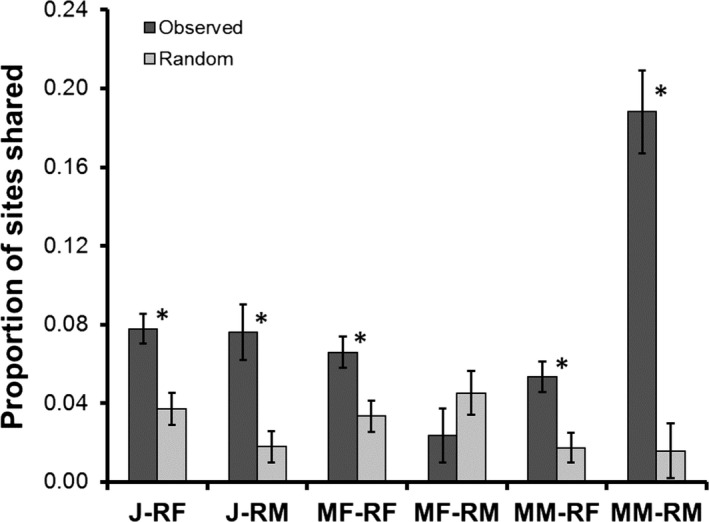
Home‐range overlap as measured by the proportion of shared sites by two or more individuals of migrant and resident *Oophaga pumilio* in a cacao plantation at La Selva, Costa Rica. Asterisks (*) indicate groups that deviated significantly from random. Groups defined as in Figure 4

Significant variation in the proportion of shared sites was observed among different migrant–resident classes (Figure 5). Juveniles shared sites with resident females and males more frequently than expected by chance (*p* < .001, *p* < .001, respectively). Migrant females shared sites with resident females more frequently than expected by chance (*p* < .001), but the proportion of shared sites with resident males did not differ from random (*p* = .052). Migrant males overlapped more frequently with both resident females and resident males than expected by random (*p* < .001, *p* < .001, respectively). An overall model testing for differences in proportion of shared sites among the six age‐sex groups was highly significant (*F*_5,1138_ = 17.60, *p* < .001). Overlap of migrant and resident males was significantly higher than all the other groups (*p* < .0001 in each case); in contrast, migrant and resident females overlapped less than all other groups (J‐RF, *p* = .001; J‐RM, *p* = .002; MF‐RF, *p* = .013; MM‐RM, *p* < .0001), except for migrant males and resident females (*p* = .076).

**Figure 6:**
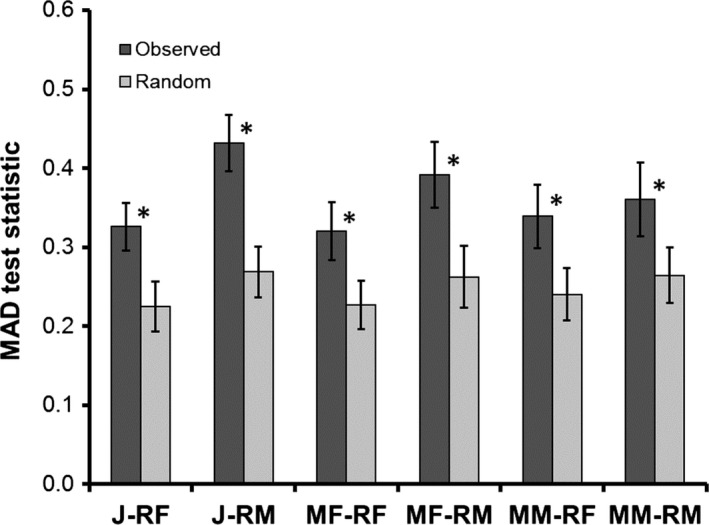
Maximum absolute deviance (MAD) test statistics from nearest‐neighbor distance distribution functions *G*(*r*) among (1) migrant and resident individuals of *Oophaga pumilio* observed in cacao plantations at La Selva Biological Station Costa Rica and (2) those generated by random. Asterisks (*) indicate when observed MAD statistics (dark gray) deviated significantly from randomly simulated data (light gray). Groups defined as in Figure 4

Maximum absolute deviation (MAD) tests of *Ĝ*(*r*) and *G*(*r*) indicated that the statistical distribution of *Ĝ*(*r*) tended toward clustered nearest‐neighbor distances relative to that expected by random (Figure 6) for each of the six migrant–resident groups (J‐RF, *p* = .0015; J‐RM, *p* < 0.0001; MF‐RF, *p* = .0023; MF‐RM, *p* = .001; MM‐RF, *p* = .0031; MM‐RM, *p* = .0066).

## 4 DISCUSSION

The conspecific attraction hypothesis predicts that, across habitats of equivalent quality, naïve juveniles and migrating adults will preferentially colonize and associate in space with preestablished adults to a greater degree than expected by chance. Thus, if *O. pumilio* are attracted to conspecifics, we predicted that, relative to random spatial patterns, migrants entering populations in a relatively homogenous cacao habitat would (1) be clumped in space with residents, (2) share sites with residents more frequently, and (3) have shorter nearest‐neighbor distances to residents. Our results provide evidence consistent with the predictions of the conspecific attraction hypothesis. For the six migrant–resident groups we analyzed, our spatial analyses described significant clumping, increased home‐range overlap, and nearest‐neighbor distances in four, five, and six of the respective groups considered.

Two nonmutually exclusive hypotheses may explain why *O. pumilio* migrating into a population as juveniles or adults may benefit from close occurrence with established resident adult females and males. First, individuals may perceive conspecifics as cues to habitat characterized by increased resources or home sites that are of sufficient quality to justify (1) a migrant's propensity to invade a site and/or (2) a resident's propensity to defend a site (conspecific cueing; Stamps, 1987). Here, migrating individuals may perceive resident females as cues to areas with access to resources sufficient for survival. In this scenario, a spatial association of migrants with residents could confer potential advantages in fitness relative to migrants occupying areas of unknown quality. Whether individuals use conspecifics as strict cues when selecting habitat merits further testing in a more rigorous experimental design (e.g., Stamps, 1987); if so, then individuals are predicted to select habitat previously occupied by a conspecific over comparable unoccupied habitat without conspecific cues.

A second hypothesis explaining why migrating *O. pumilio* are attracted to conspecifics is that attraction to conspecifics facilitates social interactions among individuals. Weygoldt (1980) was the first to describe larval provisioning by adult *O. pumilio*, and Brust (1993) described this behavior in detail and determined that larvae are obligatorily oophagous. Pröhl and Hödl (1999) found that maternal investment of *O. pumilio* is higher in females than males, that females are selective when choosing mates, and that there is significant variance in reproductive success of males (Pröhl & Hödl, 1999); together, these observations suggest that female mate choice is an important factor influencing fitness. Females also have larger home ranges than males (Donnelly, 1989b; Pröhl & Hödl, 1999), which may increase access to males when selecting mates (Murasaki, 2010). If social interactions such as female mate choice are important factors influencing the fitness of *O. pumilio*, then females with behavioral phenotypes that associate more and interact better with neighboring individuals may have greater fitness relative to individuals lacking these traits or exhibiting them to a lesser degree.

Whereas most of the migrant–resident groups analyzed showed results consistent with aggregation, such nonrandom patterns did not always manifest for migrant males+resident males or migrant females+resident males. Males are the territorial sex at La Selva (Bunnell, 1973), so migrant males may be forced to establish home ranges that avoid aggregation with resident males, an effect that might generate observed random spatial distribution patterns between those groups (Figure 4, Figure 6). However, migrant males shared sites with resident males at a significantly high rate (Figure 5). While males vigorously defend their territories by wrestling other males that enter and call within the territory, nonvocal males are not attacked (M.A. Donnelly, pers. obs.), and a recent study found evidence for a satellite tactic in which noncalling males parasitize the territories of calling males (Meuche & Pröhl, 2011). Therefore, conspecific attraction and satellite mating tactics may explain the high proportion of shared sites observed between migrant and resident males.

Nonrandom patterns of migrant females and resident males may be driven by mate choice. Females have larger home‐range areas than males, which may allow females the opportunity to carefully select mates (Donnelly, 1989b; Murasaki, 2010). Migrant females may space themselves more uniformly relative to resident males, and greater spacing relative to males would allow access to more individuals from which to choose during reproduction. This appears to be the case in our study, because spatial patterns of migrant females relative to resident males were best characterized by random in all three analyses.

Two studies to date have tested whether the conspecific attraction is a viable model explaining habitat selection of frogs. Using chemical cues from predators and conspecifics, Gonzalo et al. (2006) found no evidence that *Pelophylax perezi* respond to chemical cues of conspecifics when selecting habitat; instead, they found individuals avoided chemical cues from predators. Similarly, experimental trials of juvenile *Litoria aurea* did not document an effect of chemical conspecific cues on habitat selection, but instead found a significant effect of conspecific presence on habitat selection (Pizzatto et al., 2015). These studies suggest that conspecific attraction in frogs may be driven, at least in part, by visually mediated conspecific cues, more than chemical cues. Because *Oophaga pumilio* use visual cues during mate choice (Summers et al., 1999), visual cues from conspecifics also may provide information for individuals when selecting habitat, particularly for females who do not advertise their presence with vocalization. Acoustic cues from calling males are also likely to provide information to individuals settling habitat.

Because habitat was relatively homogenous in the plots, we assume that the observed signatures of aggregation resulted from conspecific attraction rather than habitat selection for resources. While we acknowledge that, as with any field study, variables which we did not measure may have influenced the observed patterns (e.g., spatial variance in food resources, oviposition sites, or tadpole‐rearing sites), attributes of the cacao system and the ecology of *O. pumilio* allow us to assume limited effects of confounding variables. Specifically, a predictable pattern of cacao leaf drop in the dry season and regular arrangement of trees generated a seasonal but homogenous leaf litter environment in plots, from which we can assume low variance of frog oviposition sites and foraging areas. We did not measure variance in bromeliad abundance, which may have influenced frog space use around tadpole‐rearing sites (Donnelly, 1989a); however, the cacao trees were all planted at the same time, such that we can assume the colonization of primarily epiphytic bromeliads was constrained to be uniform through plots in cacao trees.

If individuals metamorphose and enter the landscape within or near parental home ranges, then juvenile settlement might be influenced by parent recognition, either visually as a result of shared experiences during maternal provisioning or chemically through the MHC complex (Brown & Eklund, 1994; Pizzatto et al., 2015; Villinger & Waldman, 2012). If kin recognition occurs, then juveniles might exhibit preference toward settling into habitat near related individuals, which might partially confound patterns observed in this study. However, mothers are unable to directly discriminate between offspring and unrelated young during maternal provisioning (Stynoski, 2009), which suggests that kin recognition is absent in *O. pumilio* and did not influence settlement patterns of juveniles in our study.

With study limitations in mind, we still interpret our results from as preliminary support for conspecific attraction in *O. pumilio*. We believe our results represent necessary conditions of conspecific attraction; if we had not observed significant aggregation between migrants and residents, we would have been able to reject the hypothesis. However, our analysis does not provide a sufficient demonstration of conspecific attraction. While we contend our study is a productive exercise in science, a future experimental approach with rigorous controls is needed to provide a sufficient test of conspecific attraction in *O. pumilio* (e.g., Stamps, 1987).

## 5 CONCLUSIONS

The conspecific attraction hypothesis predicts that, across habitats of equivalent quality, naïve juveniles and migrating adults will preferentially colonize and associate in space with preestablished adults to a greater degree than expected by chance. Here, we use a large spatially and temporally replicated dataset to form homogenous cacao plantations at La Selva to demonstrate that juvenile and migrating adult *O. pumilio* exhibited home‐range centroids, home‐range overlap, and nearest‐neighbor distances that are consistent with necessary predictions of conspecific attraction in a field setting. Conspecific attraction may benefit migrants by providing cues to suitable home sites, reducing costs associated with prospection, and increasing potential for social interactions with conspecifics; these benefits should outweigh the negative effects of other factors associated with aggregation, such as resource competition, predator attraction, and/or pathogen transmission. This study provides support for conspecific attraction in a field setting and underscores that conspecific attraction may be a relevant mechanism for models of anuran population ecology in the Neotropics.

## AUTHOR CONTRIBUTION

BF and CG conceived the idea. MAD collected the data. BF analyzed the data. BF, MAD, and CG wrote the manuscript.
