# Supplementary material for: Spatial patterns of the frog Oophaga pumilio in a plantation system are consistent with conspecific attraction
Source: Ecol Evol. 2018 Feb 14;8(5):2880–9. doi: 10.1002/ece3.3748 (PMC5838034; doi:10.1002/ece3.3748)
Supplement: Supplementary file 1 [file ECE3-8-2880-s001.docx]

Appendix I. Candidate linear mixed-effects models explaining variation in observed abundance of *Oophaga pumilio* in cacao plantations at La Selva Biological Station, Costa Rica. The most well-supported model (ΔAIC_c_ = 0.00; model weight = 1.00) described abundance varying as a function of age-sex groups (juvenile, female, male), season, and a group-season interaction.

| Model | K | AIC_c_ | ΔAIC_c_ | Model weight | Log-likelihood |
| --- | --- | --- | --- | --- | --- |
| Group + Season + Group*Season | 17 | 308.60 | 0.00 | 1.00 | -130.02 |
| Group+Season | 9 | 336.53 | 27.93 | 0.00 | -157.46 |
| Group | 5 | 344.44 | 35.84 | 0.00 | -166.67 |
| Season | 7 | 379.85 | 71.25 | 0.00 | -181.85 |
| Null | 3 | 387.21 | 78.61 | 0.00 | -190.39 |
|  |  |  |  |  |  |
